# Multi-model inference in comparative phylogeography: an integrative approach based on multiple lines of evidence

**DOI:** 10.3389/fgene.2015.00031

**Published:** 2015-02-17

**Authors:** Rosane G. Collevatti, Levi C. Terribile, José A. F. Diniz-Filho, Matheus S. Lima-Ribeiro

**Affiliations:** ^1^Laboratório de Genética & Biodiversidade, Instituto de Ciências Biológicas, Universidade Federal de Goiás, GoiâniaBrazil; ^2^Laboratório de Macroecologia, Universidade Federal de Goiás, JataíBrazil; ^3^Departamento de Ecologia, Instituto de Ciências Biológicas, Universidade Federal de Goiás, GoiâniaBrazil

**Keywords:** biogeography, coalescence, palaeodistribution modeling, Quaternary climatic changes, vicariance

## Abstract

Comparative phylogeography has its roots in classical biogeography and, historically, relies on a pattern-based approach. Here, we present a model-based framework for comparative phylogeography. Our framework was initially developed for statistical phylogeography based on a multi-model inference approach, by coupling ecological niche modeling, coalescent simulation and direct spatio-temporal reconstruction of lineage diffusion using a relaxed random walk model. This multi-model inference framework is particularly useful to investigate the complex dynamics and current patterns in genetic diversity in response to processes operating on multiple taxonomic levels in comparative phylogeography. In addition, because of the lack, or incompleteness of fossil record, the understanding of the role of biogeographical events (vicariance and dispersal routes) in most regions worldwide is barely known. Thus, we believe that the expansion of that framework for multiple species under a comparative approach may give clues on genetic legacies in response to Quaternary climate changes and other biogeographical processes.

## INTRODUCTION

Comparative phylogeography has historically been derived from classical historical biogeography, whereby common patterns in lineage distribution within multiple taxa are often explained by vicariant events shared by all taxa (Bermingham and Moritz, 1998; Avise, 2000). However, because similar population genetic structures may arise under different demographic processes, the conventional method based on narrative descriptions and pattern interpretation derived from historical biogeography often results in dubious or indistinguishable historical demographic or vicariant processes. Thus, recovering the true demographic history of species is critical for understanding microevolutionary processes and the spatial context of lineage divergences (Knowles and Maddison, 2002), but this approach should still be expanded to the context of comparative phylogeography.

In this context, one of the major challenges in the emergent field of statistical phylogeography is to set up demographic scenarios independently of gene trees, which should help to define alternative hypotheses for temporal and geographical aspects of species dynamics and, consequently, establish relevant phylogeographical inferences (Knowles et al., 2007; Knowles, 2009; Carstens and Knowles, 2010). Recently, phylogeographers began to explore multiple lines of evidence obtained from advances in geology, paleontology, palaeopalynology, and ecology to guide choices about species dynamics across time and space, such as: (1) fossil and archeological records, including ancient DNA, as direct empirical evidence (Lagerholm et al., 2014); (2) palaeoclimatological and palaeovegetational reconstructions or general patterns of species distribution based on floristic records, as indirect evidence (e.g., Collevatti et al., 2012a,b); (3) palaeodistribution modeling, the historical extension of a model-based approach increasingly applied to macroecological and palaeobiological questions by coupling the theory of ecological niche with palaeoclimatic simulations (e.g., Carnaval and Moritz, 2008); and (4) a combination of two or more of these approaches (e.g., Metcalf et al., 2014). Due to incompleteness of the fossil record (Paul, 2009), as well as the coarse nature of the temporal and spatial resolution of palaeoecological reconstructions, the use of ecological niche models has been an accessible and efficient tool to incorporate explicit spatio-temporal information into analyses of gene trees. Such links between statistical phylogeography and macroecology are indeed an emergent approach of current phylogeographical analyses (Richards et al., 2007; Collevatti et al., 2013).

More recently, models based on Approximate Bayesian Computation (ABC) implemented in MTML-msBayes (Huang et al., 2011) have been used to test simultaneous divergence in comparative phylogeography, taking into account the stochastic variance in coalescence processes underlying multiple co-distributed lineages, thus providing general biogeographic explanations for phylogeographical patterns (e.g., Chan et al., 2011; Bell et al., 2012). Here we discuss and propose perspectives for expansion of this new framework of comparative analyses by exploring a multi-model inference approach, centered on the recent advances of statistical phylogeography (see Collevatti et al., 2012a, 2013). We first present the multi-model approach and its components and then discuss how it can be expanded to infer processes in a comparative fashion.

## COUPLING STATISTICAL PHYLOGEOGRAPHY AND MACROECOLOGICAL APPROACHES IN A MULTI-MODEL INFERENCE FRAMEWORK

The reasoning for inferring processes from a multitude of models as an alternative to null-hypothesis significance testing comes from the common need to search general explanations for observed patterns from unknown explicit causal mechanisms (Stephens et al., 2006). Multi-model inference assumes that the most effective processes causing a given observed pattern should be inferred from the best-fitted model describing empirical data (Grueber et al., 2011). However, models used in practice and the inferred processes are limited to the available information. Paul Velleman clearly synthesize this limiting factor:

“A model for data, no matter how elegant, or correctly derived, must be discarded or revised if it does not fit the data or when new or better data are found and it fails to fit them” (Velleman, 2008, p. 4).

Thus, uncertainties from multiple sources of evidence should be exhaustively explored to reach the most possible realistic and less changeable inferences, and eventually the level of uncertainty can be so high that actually many alternative models become available, allowing a multi model-based approach (Millington and Perry, 2011).

In the multi-model approach for phylogeography, uncertainties can be investigated (i) within the set of alternative demographic scenarios being considered (for instance, exploring the impact of alternative methods and climatic simulations in ecological niche modeling), (ii) during the coalescent simulation steps (when estimating genetic and dispersal parameters), and lastly (iii) at the model selection stage (given multiple available selection criteria, e.g., Akaike’s Information Criteria and likelihood estimates; Csilléry et al., 2010). Within the sub discipline of comparative phylogeography, we further propose to explore uncertainties from multiple phylogeographical hypotheses of the studied species.

## SPECIES NICHE, PALAEODISTRIBUTION MODELING, AND THE ALTERNATIVE DEMOGRAPHICAL HYPOTHESIS

**FIGURE 1:**
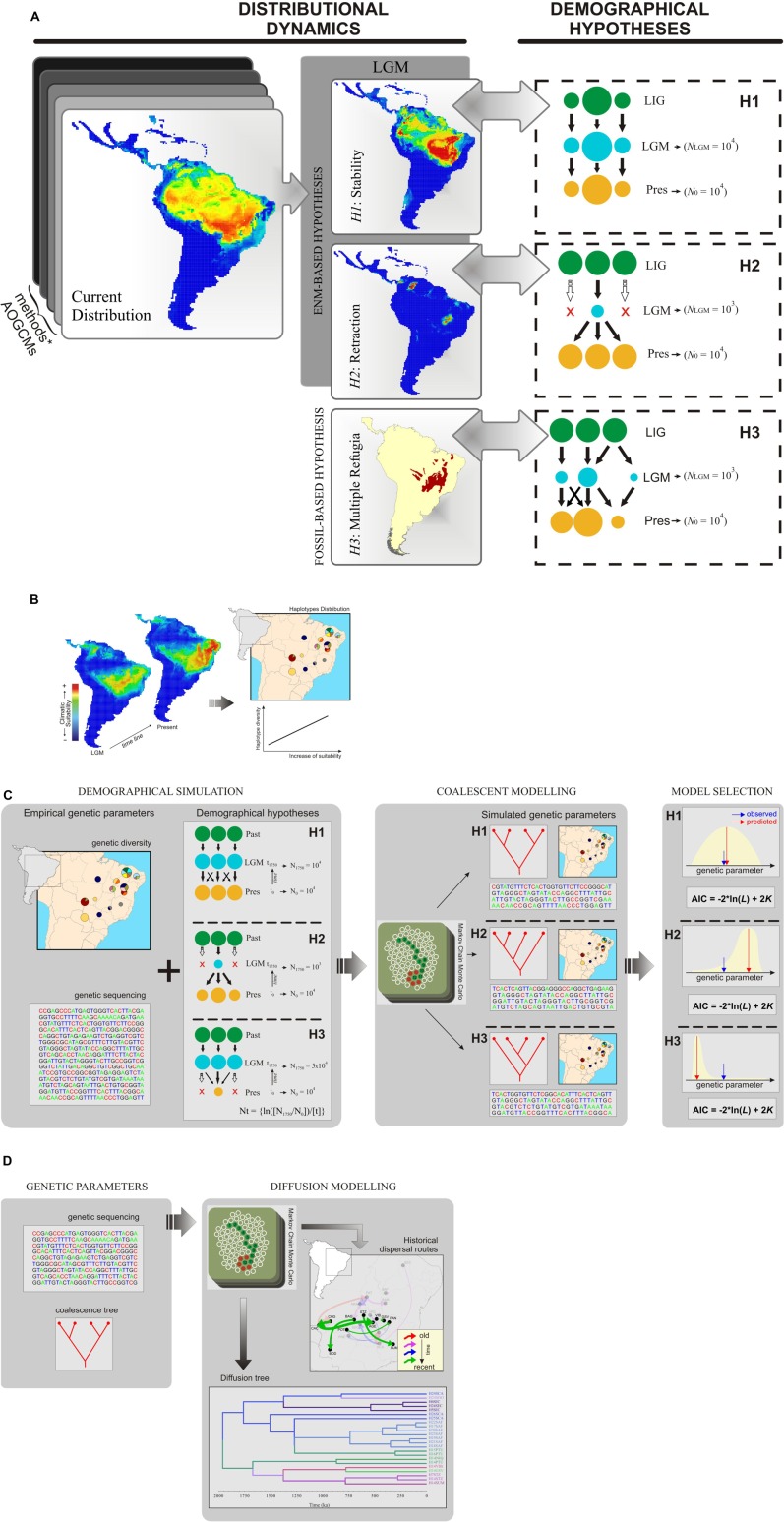
**Schematic representation for inferring phylogeographic processes using ecological niche modeling (ENM), coalescence simulation, and relaxed random walk (RRW) model. (A)** Uncertainties from ENM methods, climatic simulations (AOGCMs), and other source of evidence (e.g., fossil record) are explored to set multiple distribution dynamics through time, from which alternative demographic hypotheses are inferred. Colored circles represent demes with distinct sizes and locations through time; these demographic scenarios (demographic growth, shrink, and spatial shift) may be simulated using coalescence analyses. LIG: last interglacial (∼125,000 years ago); LGM: last glacial maximum (∼21,000 year ago); Pres: present; N_LGM_ and N_0_: effective population size at LGM and present, respectively. **(B)** An ensemble from multiple ENMs provides the environmental context to infer range shifts and perform spatial analyses considering genetic variation across populations (pie charts representing haplotype distributions). Here, maps are representing a geographic range shift of suitable areas toward the northeastern South America from LGM to present-day, which may explain the current spatial pattern of genetic diversity; i.e., increases in climatic suitability across last glacial cycle support higher haplotype diversity at present-day. **(C)** Coalescent framework used to select the most likely demographic scenario matching the empirical genetic parameters. Demographic hypotheses in a spatially explicit context through time (H1, H2, and H3; colored circles represent the population dynamics trough time) are simulated using coalescent framework to investigate their consequent population genetic structure. Models are selected from multiple criteria, such as likelihood based on posterior predictive distribution and akaike information criterion (AIC). **(D)** Relaxed random walk (RRW) modeling used to predict historical dispersal routes at the time scale of molecular sequencing data. Saving the time scaling from each model, the dispersal patterns are comparable to the ENM predictions for range shifts (see **B**). Considering the distribution map from figure, the RRW predicts intermittent dispersal routes through the time in a similar direction of range shift predicted from ENMs. The evidence from multiple models indicates that the dispersal routes predicted by RRW could be the result of climatic forcing across sequential glaciations. Moreover, RRW provides support to explore other features of population dynamics (e.g., source and sink of migrants) and biogeographic processes (e.g., dispersal barriers). Representation of Markov Chain was adapted from Professor Peter Beerli Lecture Notes (http://evolution.gs.washington.edu).

Ecological niche modeling (ENM) has allowed exploration of the geographic context of species dynamics through the past by hind casting suitable climatic conditions (an *n*-dimensional space of climatic variables) using palaeoclimatic scenarios (Martínez-Meyer et al., 2004; Nogués-Bravo, 2009). ENMs deal with the geographic context independent of gene trees, and predict species’ potential distribution over different time periods, thus providing additional information about species distribution dynamics that can be further used to set valid demographical hypotheses (Richards et al., 2007; Collevatti et al., 2013; **Figure 1A**). ENMs can actually generate multiple and independent hypotheses of species distribution history that reflect, at same time, ecological, and biogeographical realism (Collevatti et al., 2013). This is because different assumptions about the dynamics of species’ ecological niches can be explored using different ENM methods (algorithms) and palaeoclimatic simulations, which are in turn based on different modeling assumptions or types of training data (modeling uncertainty). Fossil records, when available, can be used to improve predictions of past distributions (either by providing additional information about species environmental preferences or by validating ENM predictions; Lorenzen et al., 2011; Varela et al., 2011), or to propose additional demographic hypotheses not discriminated from ENMs (**Figure 1A**).

Moreover, ENMs also provide the environmental context of population movements across geographical space through time (**Figure 1B**), as proposed by Collevatti et al. (2012b). Dispersal events may be inferred from spatially explicit analyses of population genetic structure in relation to fluctuations of climatic suitability through time (a direct measure from ENMs in areas where populations were sampled), space (habitat tracking or range shifts observed from consensual palaeodistribution as predicted by ENMs), and location of historical refugia (areas climatically suitable for the focal species throughout the time).

However, today’s practices of coupling ENMs with phylogeographic data analysis are not free of caveats. Currently, most climatic reconstructions through Atmosphere-Oceanic General Circulation Models (AOGCMs) are mainly available for short time slices, as in the case of the last 21,000 years, i.e., the time interval since the last glacial maximum (see the most recent palaeoclimatic simulations in PMIP3^1^ – and CMIP5 databases^2^). Additionally, the major lineage divergences and common phylogeographic breaks among species may have occurred earlier than this. In fact, coalescent analyses infer the time to the most recent common ancestor (TMRCA) in a time scale derived from the molecular sequence substitution rate under a particular demographic model (Kuhner, 2008). As a consequence, molecular sequences with lower mutation rates (such as chloroplast DNA for example) may lead to older divergence dating compared to the time interval of ENMs. Consequently, the predictions from ENMs and coalescent analyses would be temporarily discordant. We propose two solutions to this apparent weakness.

First, if palaeodistribution modeling is based only on climatic conditions and, most importantly, if predictions are intended to test the genetic legacy from recurrent glacial cycles, the modeler may set the distribution dynamics across the last glacial cycle (for which palaeoclimatic simulations are commonly available) and assumes analogous dynamics through the older cycles. Although separate glacial cycles have provided idiosyncratic dynamics on small temporal and geographical scales, the general pattern of intermittent glacial and interglacial periods was common throughout the Quaternary. Thus, considering broad geographical scales, it seems acceptable to assume that similar distributional dynamics have occurred across different Quaternary glaciations. In contrast, ENMs could be projected for deeper periods (e.g., last interglacial – 125,000 year – mid-Pliocene – ∼3 Million years), avoiding such important assumption, although few AOGCMs are currently available for such periods (see Stone et al., 2013, and the special issue “PlioMIP: experimental design, mid-Pliocene boundary conditions and implementation” at the journal Geoscientific Model Development, available at^3^). Thus, it is important that the assumption of analogous dynamics through time is validated by comparing the potential population movements across geographical space including the specific time slice from ENM predictions with patterns of lineage diffusion explicitly simulated by relaxed random walk (RRW) model, which encompasses deeper time that is proportional to the molecular evolution of the sequence used (Lemey et al., 2009, 2010; see also the section below). If the general patterns of population dispersal during cooling and warming phases are concordant between ENM and RRW, then assuming similar distributional dynamics across Quaternary glaciations (on broad spatio-temporal scales) is not equivocal (but see next proposal).

Second, due to recent advances on Pliocene–Pleistocene stacked estimates of isotopic globally distributed oxygen (e.g., Lisiecki and Raymo, 2005), climatic conditions may be extended backward by using the climate change between LGM and present-day to interpolate climate trends to older glacial cycles following the proportional oscillation across the deeper oxygen curve. At the same time, if predictions are at species level and comparative across multiple species, the ancestral state of the species niche may still be simulated across a phylogenetic hypothesis, so that palaeodistributions are automatically obtained at the deeper time (see example in Lawing and Polly, 2011; Rödder et al., 2013). When possible, these solutions should be preferred instead of assuming similar distribution dynamics across different Quaternary glaciations. Palaeoclimatic simulations for deeper times may improve our approach by projecting ENM predictions directly or using the temporal interpolation.

## DEMOGRAPHIC HISTORY SIMULATION AND MODEL SELECTION

To trace demographic history, demographic scenarios can be modeled, and simulated under a coalescent framework (**Figure 1C**; Kingman, 1982; see Csilléry et al., 2010 for a review of available software). Briefly, the available software runs independent simulations for each sequence region based on demographic parameters such as migration and effective population size, and under a given evolutionary model, sequence length, and mutation rate. Usually, simulation output includes genetic diversity estimates such as haplotype and nucleotide diversities or expected heterozygosity under Hardy–Weinberg equilibrium for genotypic data, number of haplotypes or alleles, parameters for neutrality, and demographic expansion tests, sequences, and genotypes.

The alternative models may be compared using several criteria (**Figure 1C**). For instance, the posterior estimates of genetic parameters for the alternative demographic scenarios can be compared with the empirical haplotype and nucleotide diversity (Csilléry et al., 2010). The likelihood of each model can be obtained from the posterior predictive distribution and the alternative models can be compared using the Akaike Information Criterion (AIC; see Burnham and Anderson, 2002). Model fitting may also be performed generating coalescent trees under each simulated demographic scenario and compared with observed coalescence time using ABC implemented in MTML-msBayes (Huang et al., 2011).

However, despite multiple lines of evidence to design alternative demographic hypotheses, spatially explicit modeling is yet to be developed. Although some advances have been made with software like SPLATCHE2 (Ray et al., 2010) and PHYLOGEOSIM 1.0^4^ (Dellicour et al., 2014), more complex palaeodistribution dynamics that can differentiate among some predictions are still unavailable. For instance, modeling demographic scenarios for range shift and range expansion is still a challenge because both scenarios may result from similar demographical dynamics (smaller effective population size in the past than in the present-day), but generate different genetic signatures due to spatial context (see Excoffier et al., 2009). Also, different range shift scenarios may generate distinct genetic signatures depending on the spatial direction of colonization of founding lineages (Excoffier et al., 2009; Waters et al., 2013).

## DIFFUSION MODEL AND CLUES TO DISPERSAL ROUTES

The lack of fossil records for most species makes understanding historical dispersal routes difficult especially in Neotropics. Thus, integrating direct spatio-temporal reconstruction of lineage dispersal may give new insights on the pathway of lineage dispersal to better understand phylogeographic patterns.

Lemey et al. (2009, 2010) proposed a Bayesian statistical approach to infer continuous phylogeographic diffusion using a RRW model, while simultaneously reconstructing the evolutionary history in time from molecular sequence data (**Figure 1D**). More specifically Lemey et al.’s (2009) approach describes the phylogeographical diffusion processes by stochastically selecting a diffusion rate scalar on each branch of the rooted phylogeny from an underlying discretized rate distribution while running a Bayesian Markov Chain Monte Carlo model. Consequently, two important advantages arise from Lemey et al.’s (2009) approach: (1) relaxing the most restrictive assumption of the standard Brownian diffusion model, and (2) infering the migration process in natural time scales (i.e., the time scale of the molecular sequence substitution process). Moreover, because this framework is based on stochastic models, it naturally accesses the uncertainties along the ancestral state reconstructions and the underlying phylogeographic process (Lemey et al., 2009, 2010), an essential component from any multi-model inference approach (Millington and Perry, 2011). We understand that this approach may be a fine complement to the static ENM predictions of population dispersal, using explicitly simulated dispersal routes in the evolutionary time scale of molecular sequences. Thus, it will become an indispensable component in a multi-model framework for phylogeographical inferences. RRW also deals with uncertainties along the ancestral state reconstructions and the underlying phylogeographical process because it is based on stochastic models (Lemey et al., 2009, 2010). The relaxed random walk model is implemented in the software BEAST 1.8.0 (Drummond and Rambaut, 2007) that analyses sequence evolution, demographic model, and lineage diffusion in space and time simultaneously, and the spatio-temporal reconstruction can be performed using SPREAD 1.0.6 (Bielejec et al., 2011). Although a promising approach, we understand that it is still necessary to find explicit methods to couple predictions from ENM and RRW.

## EXPANDING THE MULTI-MODEL FRAMEWORK FOR COMPARATIVE PHYLOGEOGRAPHY

**FIGURE 2:**
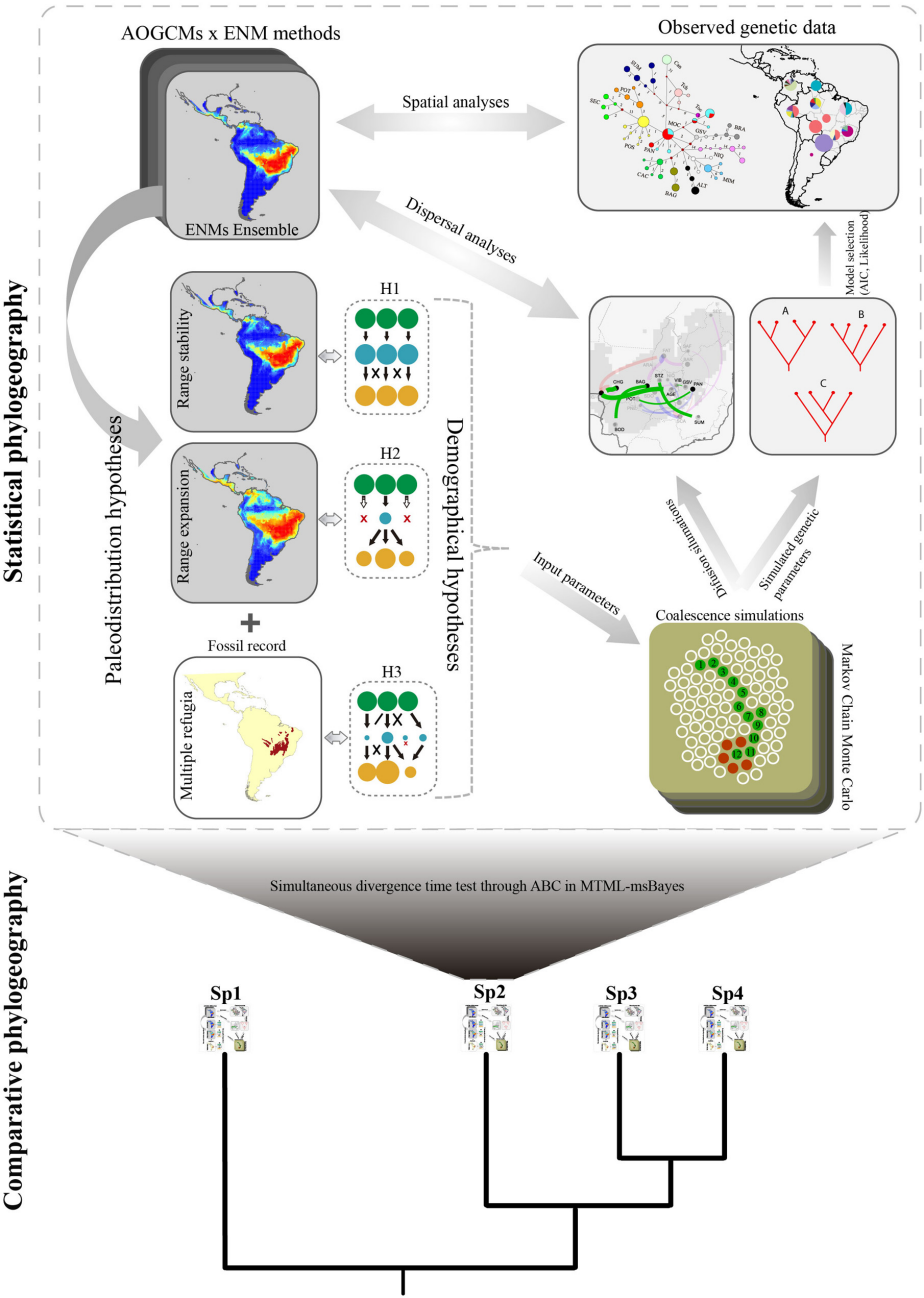
**Schematic view of the generalized framework for coupling ecological niche modeling, coalescent simulation, and diffusion model in a statistical comparative phylogeography approach.** Palaeodistribution maps resulting from ENMs are used to generate demographic hypotheses (see **Figure 1**) in a spatially explicit context through time (hypotheses H1 and H2; colored circles represent the population dynamics), according to the range shifts in species distributions in the past (e.g., range retraction or range stability). Other hypotheses may also be set based on fossil records (hypothesis H3) or *a priori* biogeographic scenario. Uncertainties in setting alternative hypotheses can be incorporated into the framework using several ENM methods and projecting distributions through different AOGCMs. In a next step, simulated coalescence structures are compared with observed data in a model selection approach, allowing selecting among the demographic hypothesis (H1, H2, or H3) the most likely to generate the current phylogeographic structure derived from molecular data. At the same time, phylogeographic diffusion models allow reconstructing colonization routes that are compared with palaeodistribution maps. Finally, this framework can be expanded into a multi-species comparative approach allowing inferring how whole assemblages responded to the interplay between climate changes, geographic barriers, and demographic processes, shaping the current patterns of species distribution, and biodiversity. Coalescent time for each species can be compared using, for instance, Approximate Bayesian Computation implemented in MTML-msBayes. Representation of Markov Chain was adapted from Professor Peter Beerli Lecture Notes (http://evolution.gs.washington.edu).

Following this reasoning of model-based inference, we propose the extension of the framework coupling ENM, coalescent simulation and the RRW model for comparative phylogeography (**Figure 2**). In a nutshell, for such an upgrade, alternative demographical hypotheses should firstly be set considering the complete range of distribution dynamics considering all analyzed species. In a similarly manner to statistical phylogeography, all uncertainties from ENMs, and other sources of palaeodistribution scenarios should be explored, such as palaeovegetation reconstruction and fossil records, to set individual species dynamics.

However, species may share common palaeodistribution dynamics or not, under ENM predictions. Due to the differences in life-history or functional traits, species may have responded differently to climate changes or other biogeographical processes (Colinvaux et al., 2000). For instance, *Tabebuia impetiginosa* (Collevatti et al., 2012a) and *Astronium urundeuva* (Caetano et al., 2008), both from seasonally dry forests in Brazil, expanded their range in response to drier and cooler periods of the glacial cycles in Neotropics, whereas in Brazilian savannas, *Caryocar brasiliense* (Collevatti et al., 2012b), and *T. aurea* (Collevatti et al., 2014) showed population retraction in multiple refugia as response to the same climatic events. The lack of common palaeodistribution dynamics, however, does not mean that different historical biogeographical process affected each species if evolutionary timing matches. Divergence timing and demographic response may be compared to better understand how Quaternary glaciations affected multiple species from distinct regions and with different traits. Concerning the comparative analyses, demographic hypotheses should be set *a priori*, and therefore biogeographical hypotheses would be particularly investigated for species with unique characters; i.e., hypotheses may be usually proposed for entire biomes or biotas (e.g., Pleistocene Arc hypothesis for Neotropical seasonally dry forests, see Prado and Gibbs, 1993) and thus may be simulated for all species from the same biome in a comparative phylogeography framework (**Figure 2**).

Consequently, the coalescent simulations based on the palaeodistribution scenarios may also be performed for all species and compared among species from the same functional group, similar ecosystems, or with similar life-histories (e.g., similar pollination and dispersal syndromes). The role of life-history or quantitative traits in shaping general phylogeographic patterns may ultimately be investigated using random or mixed effects models in meta-regression, weighting evidence by its level of uncertainty (Stanley and Jarrell, 1989). Whatever the source, the higher the uncertainty for a species (e.g., from ENM predictions) the lower is its influence to draw general phylogeographical inference under this comparative framework.

Moreover, the reconstruction of colonization routes would complement the understanding of how unique historical process affected multiple species in a broad biogeographical hypothesis (e.g., Taberlet et al., 1998). Even with a higher number of species studied, understanding the role of vicariance and dispersal routes is compromised in most regions worldwide because of the lack of direct empirical evidence from fossil records at community level. Thus, integrating direct spatio-temporal reconstruction of lineage diffusion with ecological niche modeling and coalescent simulation may indicate the pathways where multiple lineages have dispersed and their genetic legacies as a response to Quaternary climate changes and other biogeographic processes. For *T. aurea*, for instance, reconstruction of colonization routes unraveled the role of populations with higher genetic diversity at the edge of the historical climatic refugium as a source of migrants, whereas populations at the center of climatically stable areas worked usually as a sink of migrants (Collevatti et al., submitted).

In addition, integrating direct spatio-temporal reconstruction of lineage diffusion with dispersal routes predicted by the fossil record may allow validation and improvement of the lineage diffusion model. For instance, Lima et al. (2014) used the pollen fossil record of *Mauritia flexuosa,* a Neotropical swamp palm, to validate the predictions of ENM on population range shifts. The comparison of dispersal routes based on RRW models with pollen fossil records and ENM predictions can be applied to predict and validate dispersal routes during spatial population displacements.

In conclusion, along with the flexible and integrative nature of our multi-model framework in the context of the statistical phylogeography, its expansion in a comparative direction also makes it comprehensive. This aspect of our multi-model inference framework is particularly useful to investigate the complex dynamics and current patterns of genetic diversity in response to processes operating on multiple taxonomic levels as approached in comparative phylogeography.

## Conflict of Interest Statement

The authors declare that the research was conducted in the absence of any commercial or financial relationships that could be construed as a potential conflict of interest.

## Footnotes

1 https://pmip3.lsce.ipsl.fr/

2 http://cmip-pcmdi.llnl.gov/

3 http://www.geosci-model-dev.net/special_issue5.html

4 http://ebe.ulb.ac.be/ebe/Software.html
